# GDF15 promotes 5-Fluorouracil and Oxaliplatin resistance by promoting stem cell-like phenotype in colorectal cancer

**DOI:** 10.1038/s41416-026-03379-0

**Published:** 2026-05-12

**Authors:** Masahiro Hashimoto, Shoichiro Urabe, Tsuyoshi Hata, Yoshinao Chinen, Kengo Haruna, Mitsunobu Takeda, Yuki Sekido, Atsushi Hamabe, Takayuki Ogino, Norikatsu Miyoshi, Taro Tobo, Koshi Mimori, Mamoru Uemura, Hidetoshi Eguchi, Yuichiro Doki

**Affiliations:** 1 Department of Gastroenterological Surgery, The University of Osaka Graduate School of Medicine, Suita, Japan; 2 Department of Pathology, Kyushu University Beppu Hospital, Beppu, Japan; 3 Department of Surgery, Kyushu University Beppu Hospital, Beppu, Japan

**Keywords:** Colorectal cancer, Surgical oncology, Prognostic markers, Colorectal cancer

## Abstract

**Background:**

Despite recent advances in chemotherapy for colorectal cancer (CRC), chemotherapy-sensitive tumours often develop resistance to treatment, which remains a major clinical challenge. This acquired chemoresistance limits the efficacy of subsequent therapies and is associated with a poor prognosis. Therefore, this study identified the mechanisms of acquired chemoresistance in CRC and developed innovative targeted therapies.

**Methods:**

5-fluorouracil (5-FU)-and oxaliplatin (OX)-resistant CRC cells were established through long-term exposure to anticancer drugs, and RNA sequencing (RNA-seq) was performed. RNA-seq data integrated analysis from resistant cells and public single-cell RNA-seq datasets from clinical CRC samples was conducted to identify key drivers of chemoresistance. Prognostic significance was evaluated by immunohistochemical analysis of liver metastasis specimens from patients with CRC who underwent curative resection of the primary tumours.

**Results:**

Chemotherapy exposure enriched high stemness and activated TGF-β signalling. GDF15 was identified as a key molecule upregulated in both chemoresistant and high-stemness cells. Clinically, high GDF15 expression is associated with early recurrence and poor prognosis. Functional assays demonstrated that GDF15 overexpression promoted chemoresistance, stemness, and migratory capacity of CRC cells.

**Conclusions:**

GDF15 promotes chemoresistance in CRC by promoting stem cell-like properties. These findings provide insights into therapeutic strategies for overcoming acquired chemoresistance and improving outcomes.

## Introdcution

Colorectal cancer (CRC) is the third most common cancer worldwide and remains a major cause of cancer-related deaths, highlighting the need to optimize therapeutic strategies [1]. For patients undergoing chemotherapy, regimens based on fluoropyrimidines (5-FU), oxaliplatin (OX), and irinotecan, often combined with molecular targeted agents for unresectable CRC, have been shown to prolong the median overall survival [2, 3].

In the setting of adjuvant chemotherapy following curative surgery for stage III CRC, OX-based doublet regimens are widely used, achieving favourable outcomes with five-year survival rates of approximately 65–70% and recurrence rates of 20–35% [4–8]. The ACHIEVE study, which compared 3 and 6 months of adjuvant CAPOX or FOLFOX in stage III colon cancer, suggested that a 3-month therapy was as effective as a 6-month therapy, with fewer adverse effects in low-risk patients, supporting the optimization of treatment duration based on patient risk [9]. However, despite these advances in chemotherapy, the prognosis after recurrence remains poor, with the five-year survival rate after recurrence estimated at approximately 30%, and even worse in cases with unresectable metastases to the liver or lung [10–12]. The poor prognosis after recurrence is mainly due to the emergence of chemoresistant tumours that initially respond to chemotherapy, resulting in limited treatment options available once resistance develops [13, 14]. Regarding drug resistance in CRC, genetic alterations such as RAS/BRAF mutations are known to be associated with intrinsic and acquired resistance to anti-EGFR therapy, and treatment selection in clinical practice is based on these genetic profiles [15–18]. However, the mechanisms by which chemotherapy-sensitive tumours acquire resistance during treatment are complex and poorly understood [19, 20].

In recent years, advances in single-cell RNA sequencing (scRNA-seq) and spatial transcriptomics technologies have enabled a deeper understanding of cell-cell interactions and spatial architecture within tumour tissues [21, 22]. We also previously employed this approach by integrating scRNA-seq and spatial transcriptome sequence (ST-seq) analyses and found that Midkine (MDK) signalling induces immune tolerance in the adenoma-carcinoma sequence during the early stages of cancer [23]. Several recent studies have demonstrated that integrative analysis is a promising and effective approach for identifying key molecules associated with chemoresistance in gastrointestinal malignancies [24–26].

In this study, an integrated analysis of RNA sequencing data was conducted using established chemoresistant CRC cell lines and publicly available single-cell transcriptomic datasets of clinical CRC samples to clarify the underlying mechanisms associated with acquired chemoresistance and evaluate their clinical significance.

## Methods

### Patients with CRC and clinical sample collection

CRC tissues were collected from 53 patients who underwent surgery at the Department of Gastroenterological Surgery of the University of Osaka (Suita, Japan) between 2011 and 2018. All patients were diagnosed with CRC based on the following clinicopathological criteria described by the Japanese Society for Cancer of the Colon and Rectum: recurrence of liver CRC metastasis after adjuvant chemotherapy for stage III CRC. All samples were fixed in 10% formalin overnight at room temperature, processed using graded ethanol solutions, and embedded in paraffin. Specimens were used with the approval of the Ethics Committee of the Graduate School of Medicine, the University of Osaka. The present study was initiated as an opt-out study, approved by the Research Ethics Committee of the University of Osaka (approval ID: 19020-3). The opt-out policy for this study is available online (https://www.med.osaka-u.ac.jp/pub/gesurg/consent.html).　 Moreover, the Beppu score was calculated for each patient based on previously published criteria, which evaluate six preoperative clinical factors: synchronous metastasis, lymph node status of the primary tumour, number of liver metastases, maximum tumour size, presence of extrahepatic disease, and preoperative CA19-9 levels [27].

### Public datasets

scRNA-seq data were obtained from GSE178318 and spatial transcriptomic data from the spatial transcriptomic website (http://www.cancerdiversity.asia/scCRLM/). Spatial transcriptomic data of two patients (ST-P1, without preoperative chemotherapy; ST-P4, with preoperative chemotherapy; XELOX) were used. CRC RNA sequencing data from The Cancer Genome Atlas (TCGA) were downloaded from UCSC Xena (http://xena.ucsc.edu/). Gene-level transcription estimates were obtained as log2(x + 1) transformed RSEM-normalized counts.

### scRNA-seq data processing

We downloaded 3’end scRNA-seq raw count matrix data (10x Genomics) based on 140,281 cells from three patients with CRC from GSE178318 [28]. The Python package Scanpy (v1.9.3) was used for the processing. Briefly, genes detected 3 cells of the total cells, and cells with fewer than 200 expressed genes were removed and selected according to the following criteria: < 20% mitochondrial gene expression in unique molecular identifier (UMI) counts. The count matrix was normalized to 10,000 cells/cell by the total UMI count per cell, and then log-transformed by adding one and standardizing each gene using *scanpy.pp.normalized_total(target_sum* = *1e4) and scanpy.pp.log1p*. Then, highly variable genes were selected based on specific thresholds for mean expression and dispersion using *scanpy.pp.highly_variable_genes* (min_mean = 0.0125, max_mean = 3, min_disp = 0.5). Uniform Manifold Approximation and Projection (UMAP) embeddings of the latent cell states of a single cell were conducted and visualized. For the subset analysis, the UMAP was reconstructed in the extracted cell population. Major cell types were annotated by comparing canonical marker genes and differentially expressed genes (DEGs) for each cluster. The CRC stemness score was defined as the average expression of a curated colorectal cancer stem cell-related gene set and was calculated using the *scanpy.tl.score_genes* function. The gene set comprised four functional categories, selected based on previously reported surface markers of CRC stem cells : (1) the epithelial marker EPCAM; (2) the pluripotency-associated transcription factors SOX2, POU5F1, and NANOG; (3) adhesion molecules and integrins, including ALCAM, ITGB1, ITGAV, CD44, and CD24; and (4) established cancer stem cell markers, such as PROM1, ALDH1A1, LGR5, and DPP4 [29].

### Spatial transcriptomic data processing and integration of spatial transcriptomic and scRNA-seq data

Raw count matrix data were downloaded from a spatial transcriptomic dataset (10X Genomics) for 3313 spots from a patient (ST-P1) who did not receive neoadjuvant chemotherapy treatment and 3902 spots from a patient (ST-P4) who received preoperative chemotherapy [30]. The Python package Scanpy (v1.8.2) was used for processing. No spot filtering was performed for the subsequent integrated analysis. Following scRNA-seq preprocessing, genes with total counts < 10 were excluded, and genes for both scRNA-seq and spatial transcriptomics data were aligned using a raw-count matrix, which integrated 15551 genes common to both. As previously described, the estimated spatial distribution of single cells was combined with their expression profiles using the deconvolution tool, “DeepCOLOR” [23, 31]. Subsequently, the UMAP embeddings of the latent states of single cells were visualized. Spatial gene expression was examined using the imputed expression estimated after integration. The pathological clusters were clustered based on the pathological diagnosis using the Leiden clustering algorithm. Candidates for ligand–receptor communication between colocalized single cells using *deepcolor.calculate_proximal_cell_communications* with specified optional parameters (ntop_genes=1000, celltype_sample_num=500), as previously described [23].

### DEG enrichment analysis

Cluster-based detection of DEGs was performed using the Wilcoxon rank-sum test and the Benjamini–Hochberg method to correct for multiple comparisons (*scanpy.tl_rank_genes_groups*). DEGs with an adjusted *P* < 0.05 and log_2_ fold change > 1 were evaluated. Gene ontology (GO) enrichment analyses of DEGs were performed using the Python package gseapy (v1.0.4).

### Cell lines and culture

Human CRC cell lines HCT116 and SW480 were purchased from the American Type Culture Collection (Rockville, MD, USA) in 2001. Cell lines were screened for mycoplasma and maintained in Dulbecco’s modified Eagle’s medium (Sigma-Aldrich, St. Louis, MO, USA) with 10% fetal bovine serum in a humidified 5% CO2 at 37 °C.

### Generation of 5-FU and Oxaliplatin resistant cell lines

To generate 5-FU and OX-resistant cells, both HCT-116 and SW480 cells were initially exposed to 5-FU (5 μM) or OX (5 μM), and the drug concentration was gradually increased. Surviving cells were subsequently passaged and maintained under the same culture conditions for at least 20 passages, as described previously [32]. The resulting 5-FU- and OX-acclimatized cells were termed 5-FU and OX (resistant lines).

### RNA sequence (RNA-seq) analysis

RNA sequencing was performed as described previously [33]. Library preparation was performed in accordance with the manufacturer’s instructions using a TruSeq stranded mRNA sample preparation kit (Illumina, USA). Sequencing was conducted using a DNBSEQ-G400 sequencer (MGI, China) in 100-base single-read mode. After eliminating adaptor sequences using Trimmomatic version 0.38, the reads were aligned to the human reference genome (hg19) using TopHat2 version 2.1.1. Subsequently, fragments per kilobase of exons per million mapped fragments (FPKMs) were computed using Cufflinks version 2.2.1. The FPKM values were normalized to TPM (Transcripts Per Kilobase Million) values using the *fpkm2tpm* functions from the ‘iPSCpoweR’ package, to allow the comparison of gene expression between samples. Then, single-sample gene set enrichment analysis (ssGSEA) was implemented to quantitatively elucidate the stemness enrichment scores of the KEGG pathway gene sets in each CRC cell via ‘GSVA,’ R package (v1.48.3). Moreover, Gene Set Variation Analysis (GSVA) was performed on the Hallmark pathway using the ‘escape’ R package (v1.10.0). DEGs were analysed using the Wilcoxon rank-sum test and Benjamini–Hochberg method for multiple comparisons. A series of enhanced (adjusted *p* < 0.05 and log2 fold changes>1) or reduced (adjusted *p* < 0.05 and log2 fold changes < -1) genes were identified for further gene expression analysis. Based on the DEGs, Hallmark pathway enrichment analyses were performed using the Python package gseapy (v1.0.4).

### Transient overexpression of GDF15 by transfection and generation of HCT116/SW480 cells stably overexpressing GDF15

The GDF15-expressing lentiviral plasmid vector (pLV[Exp]-Puro-CMV>hGDF15[NM_004864.4]) and empty plasmid vector (pLV[Exp]-Puro-CMV > ORF_Stuffer) were purchased from Vector Builder and used as controls. For transient overexpression analysis, the plasmid vectors were transfected into CRC cells using Lipofectamine 3000 (Thermo Fisher Scientific) following the manufacturer’s protocol. To generate stably overexpressing CRC cells, lentiviruses were produced in 293TA cells using the Vira Power Lentiviral packing mix (Thermo Fisher Scientific), and the supernatant was collected 48 h after transfection. HCT116 and SW480 cells were infected with the lentiviral supernatants and selected using puromycin. Control cells were generated by transfecting cells with an empty vector containing ORF_Stuffer.

### Quantitative real-time polymerase chain reaction (qRT-PCR)

Total mRNA was isolated using the TRIzol Reagent (Invitrogen) for cell lines according to the manufacturer’s protocol. The RNA quality was assessed using a NanoDrop ND-2000 spectrophotometer (Thermo Fisher Scientific). cDNA was synthesized from 10 ng of total RNA using ReverTra Ace qPCR RT Master Mix (Toyobo Life Science) according to the manufacturer’s protocol. Quantitative PCR was performed on a Light Cycle 2.0 System (Roche Applied Science) using a Light Cycler FastStart DNA Master SYBR Green I (Roche Applied Science). The amplification conditions were as follows: initial denaturation at 95 °C for 10 min, followed by 45 cycles of denaturation at 95 °C for 10 s, annealing at 60 °C for 10 s, and extension at 72 °C for 10 s. The mRNA levels were normalized to ACTB mRNA as an internal control. Gene expression was quantified using specific oligonucleotide primers. The primers used for qRT-PCR are listed in Supplementary Table S1. Data are representative of at least three independent experiments.

### Immunohistochemical staining

Tissue sections of 4-μm thickness were prepared from paraffin-embedded blocks, and immunostaining was performed, as previously described [33]. The slides were incubated overnight at 4 °C with anti-rabbit polyclonal antibody against GDF15 (1:400, 27455-1-AP, Proteintech). To evaluate GDF15 expression in tumour cells, the cytoplasmic staining intensity of normal liver cells was used as a positive control. Staining intensity in the tumour cytoplasm was scored as follows: +3 for cytoplasmic staining stronger than the positive control, +2 for staining as intense as the positive control, +1 for staining weaker than the positive control, and 0 for unstained cytoplasm.

### Proliferation assays

Transfected cells were seeded at a density of 2000 cells per well in 96-well plates. After culturing with exposure to 5-FU or OX (0, 2.5, 5.0, 10, 20, and 40 μM) for 72 h, cell viability was determined using Cell Counting Kit-8 (Dojindo Molecular Technologies, Inc., Kumamoto, Japan).

### Sphere formation assays

The sphere-formation assay was performed as previously described [34]. The cells were seeded in 96-well ultra-low attachment plates (Corning Inc. Corning) at a density of 200 cells/well. Cells were cultured in DMEM/F-12 serum-free medium supplemented with 20 ng/mL epithelial growth factor, 10 ng/mL fibroblast growth factor-2, and 100 μg/mL penicillin G at 37 °C in a humidified atmosphere of 95% air and 5% CO2. The number of spheres ≥100 µm in all wells was counted, and differences in the average number per well 2 weeks after seeding were evaluated.

### Flow cytometric analysis

Flow cytometry was performed using a BD FACS Canto II flow cytometer (BD Biosciences, San Jose, CA, USA). Doublets were excluded using FSC-A/FSC-H and SSC-A/SSC-H gating, and dead cells were removed using 7-AAD (BD Pharmingen, BD Biosciences, Franklin Lakes, NJ, USA). Cells were stained with PE-labelled mouse anti-human CD44 (BD Pharmingen) and Brilliant Violet 421-labelled anti-human CD133 (BioLegend, San Diego, CA, USA) according to the manufacturer’s instructions. Gating thresholds for CD44 and CD133 were determined based on the fluorescence signals from unstained parental HCT116 cells to define the negative population. The same gating strategy and identical instrument settings were applied to all samples. Data were analysed using FlowJo software v10.2 (BD Biosciences).

### Wound-healing assays

The cells were seeded in 6-well plates and grown to 80% confluence. Linear wounds were created by scraping monolayer cells with a 200 μL pipette tip, and non-adherent cells were washed off with medium. At 0, 24, and 96 h after the creation of wounds, cell migration was observed, and images were captured with a 4x objective using a phase-contrast microscope. Cell migration was determined based on the reduction in wound width measured at five independent locations using ImageJ software.

### Western blot analysis

Western blotting was performed as follows. Each sample was homogenized in 1 ml of lysis buffer [50 mM Tris (pH 8.0), 150 mM NaCl, and 0.5% NP40] with protease inhibitors (1 mM phenylmethylsulfonyl fluoride, 10 μg/ml aprotinin, and 10 μg/ml leupeptin). The homogenates were centrifuged at 14,000 rpm for 20 min at 4 °C. The resulting supernatants were collected, and the total protein concentration was determined using the Bradford protein assay (Bio-Rad, Hercules, CA, USA). To analyse lysates on the JESS Simple Western™ instrument (ProteinSimple^®^, Bio-Techne, Minneapolis, MN, USA), samples were diluted to total protein concentrations of 1 µg/µL in 1x fluorescent master mix (EZ standard pack I; ProteinSimple^®^), and 5 µL was added per well. Primary antibodies used in this study are listed in Supplementary Table S2. All antibodies were diluted 1:40 in Ab diluent (Bio-Techne), and 10 µL was added per well. The secondary Ab (anti-rabbit HRP) and enhanced chemiluminescence reagents were used according to the kit’s instructions (anti-rabbit detection module chemiluminescence; ProteinSimple^®^, Bio-Techne). The Ab diluent, washing buffer, plates, and capillary cartridges used were derived from the 12 to 230 kDa separation module (ProteinSimple^®^, Bio-Techne). All procedures were performed according to the manufacturer’s instructions. JESS Simple Western™ data were analysed using Compass for Simple Western software. Images from the high-dynamic range 4.0 were used for analysis, and the peaks were automatically detected. Both the peak height and area were analysed.

### Statistical analysis

The statistical analyses were performed using R software v4.2.0 and Python v3.9.16. Associations between variables were analysed using Welch’s *t*-test and the Wilcoxon rank-sum test. Overall survival (OS) and recurrence-free survival (RFS) curves were plotted using the Kaplan–Meier method and compared using the log-rank test. Univariate and multivariate analyses were performed using the Cox proportional hazards model to identify the independent variables predictive of OS and RFS. A two-sided *P*< 0.05 was deemed statistically significant.

## Results

### 5-FU and OX-resistant CRC cells promote cancer stemness

**Fig. 1:**
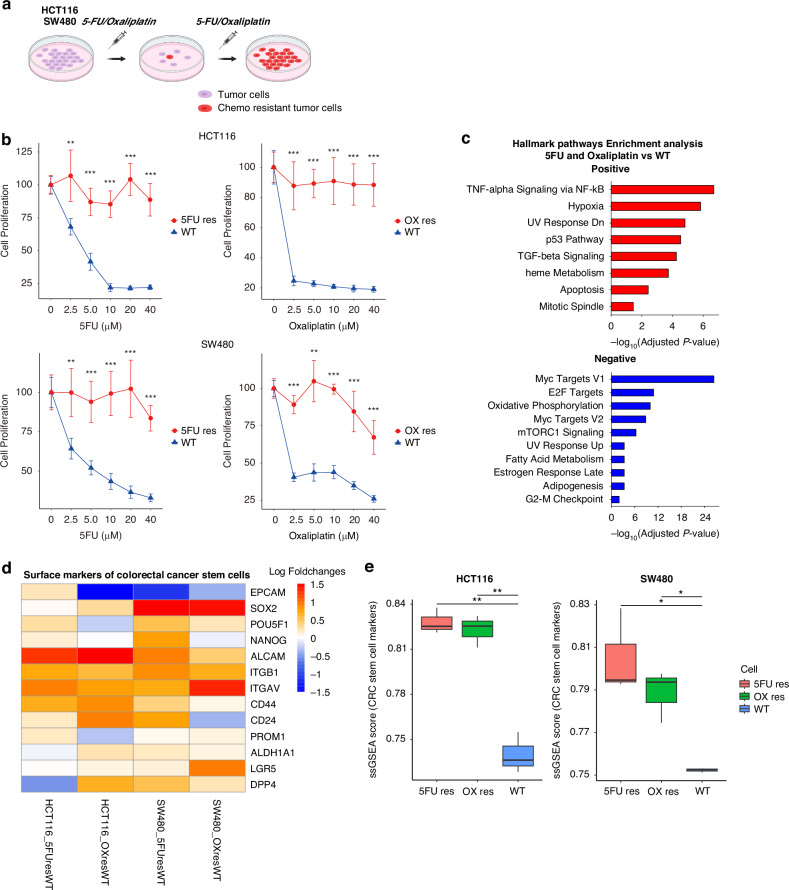
Generation and characteristics of 5-FU and Oxaliplatin resistant (5FU res, OX res) colorectal cancer cells. **a** Schematic representation of the generation of 5-FU- and oxaliplatin-resistant colorectal cancer cells. **b** Proliferation assay analysis of CRC cells with 5-FU/Oxaliplatin resistant versus control cells (*n* = 5). **c** Hallmark pathway enrichment analysis of CRC cells with 5-FU/Oxaliplatin resistant versus control. **d** Heatmap of the expression of surface markers in colorectal cancer stem cells with 5-FU/Oxaliplatin resistant versus the control, categorized by their biological functions. **e** Comparison of enrichment scores for cancer stem cell-related genes in KEGG pathways. *P < 0.05, **P < 0.01, ***P < 0.001; *P* values were determined using Welch’s t-test.

HCT116 and SW480 cells were used to generate 5-FU- and OX-resistant cells after long-term exposure to these drugs (Fig. 1a). Both cell lines showed increased resistance to 5-FU and OX in cell viability assays (Fig. 1b). To characterize 5-FU- and OX-resistant cells, RNA-seq and an enrichment analysis of hallmark pathways were performed and conducted, respectively (Supplementary Fig. 1). These resistant cells were positively enriched in the TGF-β signalling pathway, which has been reported as a regulator of cancer stemness and metastasis [35]. Moreover, they were negatively enriched in Myc pathway, which has been shown to directly interact with SMAD2/3 to inhibit TGF-β signalling [36] (Fig. 1c). Next, the expression of surface markers on CRC stem cells was examined [29] and found that many stem cell marker genes were highly expressed in these drug-resistant cells (Fig. 1d). Based on ssGSEA enrichment scores of stemness in the KEGG pathways [37], significantly increased stemness was observed in both cell lines (Fig. 1e). These results suggest that TGF-β signalling and cancer stemness are upregulated in 5-FU and OX-resistant cells.

### Epithelial cells in the chemotherapy cases of CRC increase cancer stemness

**Fig. 2:**
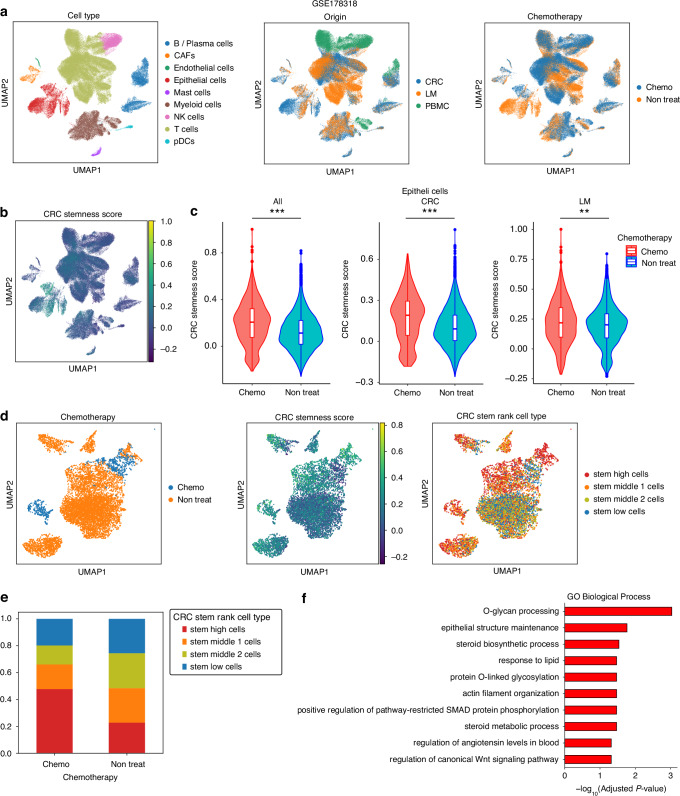
Analysis of the association between chemotherapy and cancer stemness in single-cell colorectal cancer (CRC) data. **a** Uniform Manifold Approximation and Projection (UMAP) of cell types in all cells (left), tissues (middle), and treatments (right). Tissue origins consisted of primary CRC, liver metastasis (LM), and peripheral blood mononuclear cells (PBMCs). **b** CRC stemness scores in UMAP representations. **c** Enrichment analysis of the CRC stemness score in epithelial cells treated with chemotherapy versus untreated cells. **d** UMAP of epithelial cells in treatments (left), CRC stemness scores (middle), and CRC stemness ranking types (right). **e** Proportion of cells classified based on stemness scores. **f** Gene Ontology (GO) enrichment analysis of biological processes comparing high-stemness epithelial cells with other epithelial cells.

The relationship between chemotherapy and cancer stemness in CRC tissues was evaluated using publicly available CRC scRNA-seq data from six patients from GSE178318 (three preoperative chemotherapy cases and three untreated cases) [28]. The scRNA-seq data contained 133,132 cells and nine cell types were annotated with marker genes (Fig. 2a). We performed enrichment analysis using CRC stem cell marker genes for all cell types (Fig. 2b). In epithelial cells, the CRC stemness scores were significantly higher in the chemotherapy cohort than in the non-treated cohort for all cells of primary CRC and liver metastasis origins (Fig. 2c). To characterize the stemness of the epithelial cells, we classified them into four groups based on the CRC stemness score (Fig. 2d, Supplementary Fig. 2a). In both the primary CRC and liver metastasis cases, the chemotherapy cohort had a higher proportion of cells with high stemness scores than the untreated cohort (Fig. 2e, Supplementary Fig. 2b). Furthermore, enrichment analysis was performed on the GO biological processes for cells with a high stemness score. GO analysis revealed that SMAD protein phosphorylation, which promotes TGF-β signalling, was positively enriched (Fig. 2f). These analyses of CRC clinical samples indicated that the chemotherapy cohort had a high proportion of high stem cells and was positively associated with TGF-β signal pathways. These results are consistent with our findings in chemoresistant CRC cell lines.

### Identification of GDF15 involved in cancer stemness in CRC cells

**Fig. 3:**
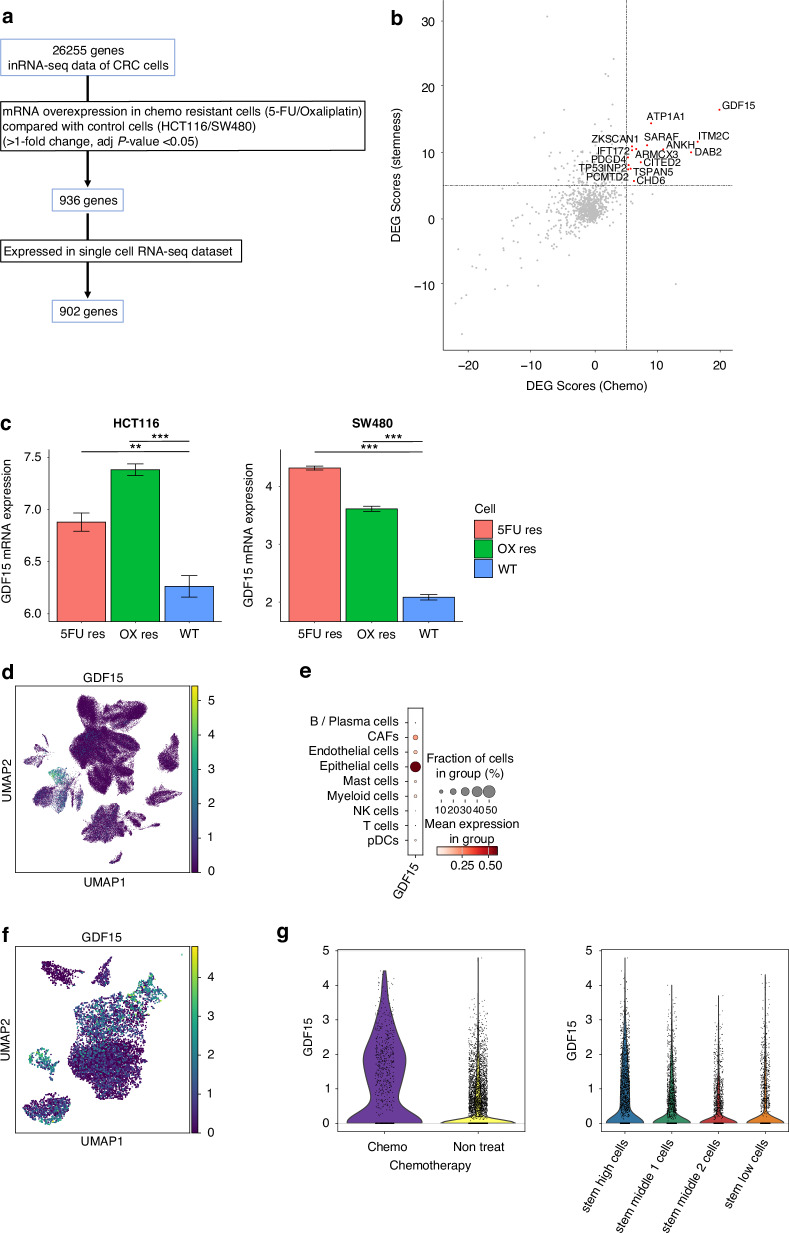
High expression of GDF15 in both 5-FU/Oxaliplatin resistant colorectal cancer cells and single-cell colorectal cancer (CRC) data. **a** Identification of highly expressed genes in the RNA-seq data of CRC cells and single-cell RNA-seq data. **b** Differentially expressed genes in the high stemness cell and chemotherapy groups. **c** GDF15 mRNA expression in the RNA-seq data of 5-FU/Oxaliplatin resistant and control cells. **d** Uniform Manifold Approximation and Projection (UMAP) of GDF15 expression in all cells. **e** Dot plot of the expression and proportion of GDF15 per cell subtype in different cell types; the circle size represents the cell proportion. **f** UMAP of GDF15 expression in primary CRC epithelial cells. **g** Violin plot of GDF15 expression levels in primary CRC epithelial cells based on chemotherapy status (left) and CRC stemness rank types (right).

Moreover, we identified novel driver genes associated with chemotherapy and cancer stemness. From 26,255 genes in the RNA-seq data of CRC cell lines, 902 candidate genes were extracted, which satisfied the following two criteria: (1) mRNA overexpression in chemoresistant cells compared to control cells (adjusted *p* < 0.05, log2 fold changes>1), and (2) genes common to the scRNA-seq data (Fig. 3a). Among these 902 genes, the growth differentiation factor 15 (GDF15) was identified as the most highly expressed gene in the chemotherapy cohort and in epithelial cells with a high stemness score (Fig. 3b). GDF15 is a stress-induced cytokine, a member of TGF-β superfamily, and is highly expressed during tissue injury, oxidative stress, and cancer [38]. RNA-seq analysis of resistant cells showed that GDF15 mRNA levels were significantly higher in 5-FU- and OX-resistant cells than in control cells for both cell lines (Fig. 3c). Moreover, scRNA-seq analysis showed that GDF15 was highly expressed in the epithelial cells (Fig. 3d, e). In both primary CRC and liver metastasis cases, epithelial cells of the chemotherapy cohort and high-stemness cells showed high GDF15 expression (Fig. 3f, g, Supplementary Fig. 3). These results suggested that GDF15, which is involved in the TGF-β signalling, may be associated with high stemness after chemotherapy.

### Spatial transcriptomic analysis integrated with scRNA-seq of CRC tissues

**Fig. 4:**
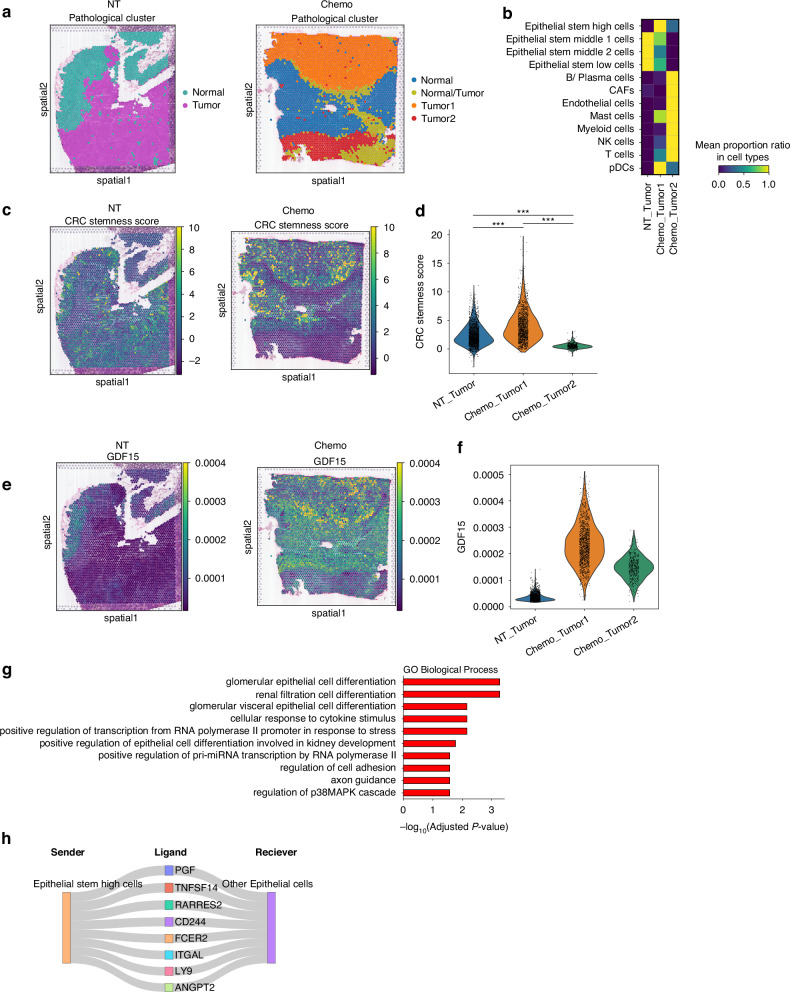
Integrative analysis of spatial transcriptomic and single-cell analysis revealing a positive correlation between GDF15 expression and colorectal cancer (CRC) stemness. **a** Spatial visualization of pathological clusters classified using the Leiden algorithm and pathological diagnosis in untreated (NT) and chemotherapy (Chemo) CRC sample slides. **b** Heatmap of the mean proportion values per spatial pathological diagnosis of each cell type in the tumour areas. **c** Spatial distribution of CRC stemness scores in untreated and chemotherapy-treated CRC sample slides. **d** Violin plot representing the CRC stemness score in tumour areas of untreated and chemotherapy-treated CRC samples. ***P < 0.001. *P* values were determined using the Wilcoxon rank-sum test. **e** Spatial distribution of GDF15 expression in untreated and chemotherapy-treated CRC sample slides. **f** Violin plot representing GDF15 expression in tumour areas of untreated and chemotherapy-treated CRC samples. **g** Gene Ontology (GO) enrichment analysis of biological processes comparing chemotherapy tumour areas where cancer cells remained (Chemo_Tumor1) to other areas. **h** Ligand activity initiated from epithelial stem cells to other epithelial cells. The widths of the lines correspond to ligand activity scores.

To examine the relationship between spatial GDF15 expression and chemotherapy in CRC tissues, spatial transcriptomic analysis was performed at the single-cell level using previously published CRC data [30] (Supplementary Fig. 4a–b). After integration analysis, the pathological clusters were clustered based on pathological diagnosis and spatial distribution of gene expression (Fig. 4a). For chemotherapy-treated CRC tissues, clusters were classified into residual tumour areas and chemotherapy response areas. Consistent with the scRNA-seq analysis, the number of high-stemness epithelial cells was high in the residual tumour areas of chemotherapy-treated CRC tissues (Fig. 4b and Supplementary Fig. 4d). Regarding the spatial distribution of CRC stemness scores, the residual tumour areas of chemotherapy-treated CRC tissues were significantly larger than those of the other tumour areas (Fig. 4c, d). GDF15 expression was assessed in CRC tissues, and the residual tumour areas of chemotherapy-treated CRC tissues were found to have highly expressed GDF15, which is consistent with the spatial distribution of high CRC stemness scores (Fig. 4e, f). GO analysis revealed that the cellular response to cytokine stimuli and stress processes was enriched in the tumour residual areas of chemotherapy-treated CRC tissues (Fig. 4g). Furthermore, we predicted ligand-mediated intercellular communication from high-stemness epithelial cells to other epithelial cells by combining data on colocalization scores, gene expression at the single-cell level, and ligand-receptor relationships. In particular, PGF and ANGPT2 were found to promote tumour cell migration (Fig. 4h) [39, 40]. These integrative spatial analyses indicated that GDF15 expression was positively correlated with CRC stemness in residual tumour regions after chemotherapy. These results are consistent with our findings in chemotherapy-resistant CRC cells and scRNA-seq analysis.

### GDF15 promotes chemotherapy resistance and stemness in CRC cells

**Fig. 5:**
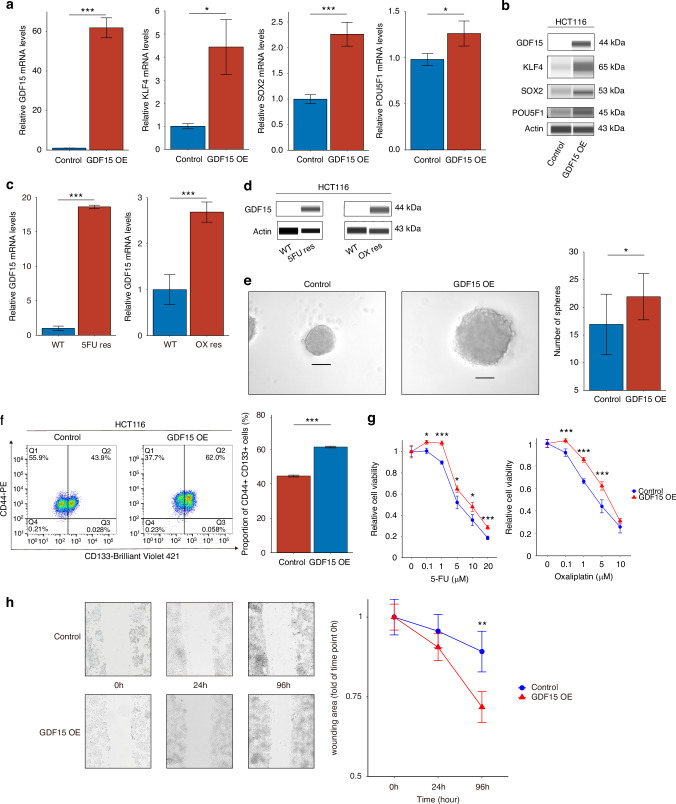
In vitro analysis of cancer stemness and chemotherapy resistance in GDF15 overexpression cells. **a** GDF15, KLF4, SOX2, and POU5F1 mRNA expression in HCT116 cells with GDF15 stably overexpression and in control colorectal cancer (CRC) cells. *P < 0.05, ***P < 0.001; *P* values were determined using Welch’s *t*-test. **b** Protein expression using western blot analysis (WB) for GDF15, KLF4, SOX2, POU5F1, and Actin in GDF15 stably overexpression and CRC cells. **c** GDF15 mRNA expression according to RT-qPCR in 5-FU/Oxaliplatin resistant and control HCT116 cells. ***P < 0.001; *P* values were determined using Welch’s t-test. **d** Protein expression using WB for GDF15 and Actin in 5-FU/Oxaliplatin resistant and control HCT116 cells. **e** Sphere formation assay of CRC cells showing induction of sphere-forming activity with stable GDF15 overexpression and control CRC cells (*n* = 12). Scale bars, 100 μm; original magnification, ×200. *P < 0.05; *P* values were determined using Welch’s t-test. **f** Flow cytometric analysis of CD44 and CD133 expression in HCT116 cells with stable GDF15 overexpression and control CRC cells (*n* = 6). ***P < 0.001; *P*-values were determined using Welch’s *t*-test. **g** Proliferation assay of CRC cells with stable GDF15 overexpression and control CRC cells treated with 5-FU (left) and oxaliplatin (right) (*n* = 5). *P < 0.05, ***P < 0.001; *P* values were determined using Welch’s *t*-test. **h** Wound healing assay to determine cell migration. Wound-healing assays were performed at 0, 24, and 96 h in HCT116 cells with GDF15 stably overexpression and in control CRC cells (*n* = 5). Representative phase-contrast microscopy images showing the area covered by the cells at 0, 24, and 96 h after wounding. Original magnification: 10 × (left). Cell migration was determined by the rate of cells moving towards the scratched area over time using the ImageJ software. **P < 0.01; *P* values were determined using Welch’s t-test.

GDF15 was assumed to play a significant role in inducing 5-FU and OX resistance and CRC cancer stemness. To validate our hypothesis, the HCT116 cell line was used for GDF15 overexpression analysis. The mRNA levels of KLF4, SOX2, POU5F1, ITGAV, and ITGAB1 were significantly increased by GDF15 overexpression in CRC cells (Fig. 5a, Supplementary Fig. 5). Western blot analysis showed that the expression of KLF4, SOX2, and POU5F1 increased in GDF15 overexpressing cells (Fig. 5b). GDF15 expression was also elevated in 5-FU- and OX-resistant cells (Fig. 5c, d). Furthermore, to determine the association between GDF15 and stemness, a sphere formation assay was performed, which revealed that the number of spheroids significantly increased in stable GDF15 overexpressing cells (Fig. 5e). Consistently, flow cytometry revealed a significant increase in the proportion of CD44⁺CD133⁺ cells in GDF15-overexpressing cells compared to that in control cells (Fig. 5f), further supporting enhanced cancer stemness. Next, a cell viability analysis was performed using HCT116 cells stably overexpressing GDF15. Compared to control cells, GDF15 overexpressing cells exhibited reduced sensitivity to 5-FU and OX (Fig. 5g). Finally, wound healing assays were performed using stable GDF15 overexpressing cells, which revealed that these cells significantly enhanced cell migration (Fig. 5h). These results highlight the critical role of GDF15 in inducing stemness and its involvement in chemotherapy resistance to 5-FU and OX, and epithelial-mesenchymal transition (EMT).

### Clinical significance of GDF15 expression levels in CRC cases after adjuvant chemotherapy

**Fig. 6:**
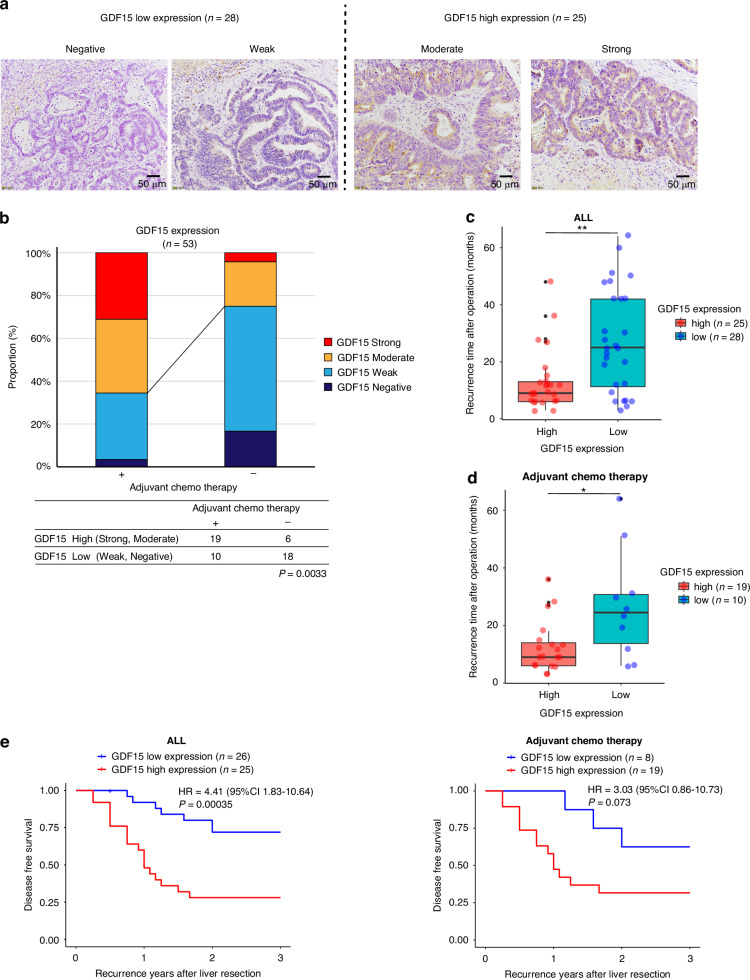
Clinical significance of GDF15 expression in human colorectal cancer (CRC) liver recurrence metastasis after primary CRC surgery. **a** Immunostaining for GDF15 in CRC liver recurrence and metastasis cases. **b** Proportion of cases classified based on GDF15 expression in adjuvant chemotherapy samples. *P*-values were determined using the chi-square test. Violin plot representing recurrence time after primary CRC surgery with high and low GDF15 expression in all cases (**c**) and adjuvant chemotherapy cases (**d**). *P < 0.05, **P < 0.01; *P*-values were determined using Welch’s *t*-test. **e** Disease-free survival rate after R0 liver recurrence resection in patients with CRC according to GDF15 expression in all cases (left) and adjuvant chemotherapy cases (right). Disease-free survival was estimated using the Kaplan–Meier method, and survival curves were compared using the log-rank test. CI confidence interval, HR hazard ratio.

GDF15, which is highly expressed in various cancer types, has potential as a diagnostic and prognostic biomarker for cancer [38, 41]. The association between GDF15 expression and prognosis was investigated using immunohistochemical analysis of CRC liver metastatic tissues after primary CRC resection in 53 patients with stage II and III CRC. GDF15 expression was observed in the membrane and/or cytoplasm, with varying degrees of staining intensity: negative staining in 5 cases, weak staining in 23 cases, moderate staining in 15 cases, and strong staining in 10 cases (Fig. 6a). GDF15 expression in adjuvant chemotherapy cases after primary CRC resection was higher than that in untreated cases (Fig. 6b). Our cohort data showed that high GDF15 expression was significantly associated with recurrence time after primary CRC resection in all cases and in adjuvant chemotherapy cases (Fig. 6c, d, Supplementary Fig. 6). In the clinicopathological analysis, high GDF15 expression was associated with an elevated frequency of adjuvant chemotherapy, lymph node metastasis in the primary tumour, and higher Beppu scores (Supplementary Table S3). Preoperative chemotherapy response was also evaluated; however, no difference was observed between the high- and low-GDF15 groups (Supplementary Table S4). Furthermore, patients with high GDF15 expression had significantly poorer disease-free survival after R0 resection of liver metastases (Fig. 6e). In the multivariate analysis, the Beppu score (*p* = 0.030) and GDF15 expression (*p* = 0.0064) emerged as independent prognostic factors for recurrence-free survival in patients with CRC who underwent R0 liver metastasis resection (Table 1). These findings suggested that GDF15 may act as an important mediator of post-chemotherapy recurrence after primary CRC resection, given its association with poor prognoses.

**Table 1:** Univariate and multivariate analysis of clinicopathological factors affecting the recurrence survival in patients with colorectal cancer liver metastasis after R0 resection.

Variables	HR	Univariate	*P*-value	HR	Multivariate	*P*-value
		(95% CI)			(95% CI)	
Age(≥65 y/<65 y)	1.20	0.54–2.67	0.66			
Gender (male/female)	2.32	0.93–5.83	0.072			
Adjuvant chemotherapy (+/−)	1.86	0.82–4.21	0.14			
Size (>2 cm/≤2 cm)	0.88	0.40–1.96	0.76			
Number (>1/1)	2.04	0.90–4.62	0.088	1.12	0.38–3.31	0.84
Tumour location (rectum/colon)	0.72	0.29–1.80	0.48			
Histological type in liver metastasis (muc,por,sig /pap,tub)	0.56	0.08–4.12	0.57			
T stage in primary tumour (is,1,2/3,4)	1.27	0.48–3.39	0.63			
Lymph node metastasis in primary surgery (+/−)	2.25	0.99–5.11	0.052	0.46	0.14–1.58	0.22
CEA in liver metastasis resection (>5 ng/mL/≤5 ng/mL)	1.49	0.66–3.37	0.34			
CA19-9 in liver metastasis resection (>37 U/mL/≤37 U/mL)	2.12	0.95–4.73	0.068	1.67	0.68–4.10	0.27
Beppu score (≥7/ < 7)	4.21	1.90–9.37	0.00042	3.67	1.13–11.94	0.030
GDF15 expression (high/low)	4.41	1.83–10.64	0.00097	4.62	1.54–13.85	0.0064

*CI* confidential interval, *CRC* colorectal cancer, *HR* hazard ratio, *muc* mucinous adenocarcinoma, *por* poorly differentiated adenocarcinoma, *sig* signet ring carcinoma, *pap* papillary adenocarcinoma, *tub* tubular adenocarcinoma.

## Discussion

Chemoresistance in CRC can be classified as intrinsic or acquired [42, 43]. Intrinsic resistance is often associated with genetic alterations and diversification of signalling pathways, such as RAS mutations, which are known to cause resistance to anti-EGFR therapies [44, 45]. In clinical practice, these genetic profiles are used to identify molecular targeted therapies [15–18]. Acquired resistance develops when tumours that initially respond to treatment gradually become resistant. Several mechanisms have been implicated in this process, including activation of drug efflux transporters, alterations in DNA repair and apoptosis pathways, and modifications in the tumour microenvironment, and these mechanisms may act together and contribute to the development of cross-resistance [46, 47].

Traditionally, resistance mechanisms have been studied by long-term exposure of cancer cells to anticancer drugs to generate resistant cells [48]. There are reports in which anticancer drugs are administered according to clinical dosing schedules to evaluate the process of resistance acquisition along with associated cytological and molecular biological changes [49–51]. Our laboratory has also employed these methodologies and elucidated the mechanisms underlying chemoresistance and cancer stemness mediated by CCNG2 in pancreatic cancer, the induction of EMT and chemoresistance via crosstalk between IL-6 and TGF-β1 in biliary tract cancer, and the synergistic antitumor effect of combination therapy of gemcitabine with an anti-FGFR inhibitor in FGF-activating cholangiocarcinoma [52–54]. This study established CRC cell lines resistant to 5-FU and OX based on postoperative chemotherapy timelines. RNA-seq analysis of these resistant cells revealed enrichment of the TGF-β signalling pathway. TGF-β signalling has been widely associated with cancer stem cells (CSCs) and is considered a regulator of stemness [35]. TGF-β has been reported to act as a tumour suppressor by transmitting signals via SMAD proteins, but once cancer is formed, it promotes invasion and metastasis, and is also involved in EMT and the maintenance of CSCs [55]. CSCs have been reported to contribute to chemoresistance [56–61]. Increased expression of stem cell markers in 5-FU-resistant cells has been reported, suggesting a link between drug resistance and cancer stemness [62]. Based on these reports, we focused on the association between chemoresistance and properties of CSCs in CRC cells.

Advancements in scRNA-seq and ST-seq have enabled detailed analysis of the spatial distribution and gene expression profiles of individual cells, revealing cell-cell interactions within specific tumours [21, 22]. Previously, scRNA-seq and ST-seq data were integrated using a deep learning framework to analyse cell colocalization and reported that MDK signalling induces immune tolerance in the adenoma-carcinoma sequence during the early stages of cancer [23]. In this study, integrated analysis of publicly available scRNA-seq and ST data revealed increased stemness scores in chemotherapy-treated samples, and enrichment of TGF-β/SMAD signalling pathways in high-stemness cells [28, 30, 31]. Additionally, through RNA-seq of our chemotherapy-resistant CRC cell lines and clinical single-cell data, GDF15 (Growth Differentiation Factor 15) was identified as a gene associated with acquired chemoresistance.

GDF15, a member of the TGF-β superfamily, is a cytokine released under inflammatory stress [63]. It is expressed at low levels in normal colon tissue but is elevated in tumours under hypoxic or stress conditions [38, 64]. Its role in CRC progression, metastasis, and chemoresistance has attracted increasing attention in recent years [65]. Previous studies have reported that elevated GDF15 expression promotes EMT and metastasis and is associated with recurrence rates in stage I–III CRC [66, 67]. GDF15 upregulation has also been implicated in OX resistance [68]. Furthermore, an association between GDF15 and stemness has been reported in breast cancer, multiple myeloma, and glioma [69–71]. Based on these observations, we focused on the possibility that GDF15 expression in CRC contributes to chemoresistance via the acquisition of cancer stemness. In the present study, GDF15 expression was found to be upregulated in chemoresistant CRC cells, suggesting that long-term exposure to anticancer agents induces GDF15 expression as a stress response. In our in vitro experiments, GDF15-overexpressing CRC cells showed increased resistance to 5-FU and OX, upregulation of stem cell markers,　enhanced sphere-forming ability, and an increased proportion of CD44⁺CD133⁺ cells, indicating that GDF15 expression is associated with chemoresistance and cancer stemness. Moreover, we found significantly higher GDF15 expression in the adjuvant chemotherapy group when comparing GDF15 expression in liver metastases between the adjuvant chemotherapy and non-chemotherapy groups using clinical samples from patients with CRC with liver metastasis. In metastatic CRC, there have been reports of altered apoptotic activity and proliferative capacity compared to primary tumours, with hypoxia acting as an aggravating factor [72, 73]. The involvement of EMT in metastasis has been well documented, and its association with TGF-β signalling has also been suggested [74, 75]. Zhan et al. indicated that GDF15–TGFBR2 signalling between cancer stem-like cells and myofibroblast-like CAFs promotes chemoresistance in liver metastases after 5-FU treatment [76]. Taken together, these findings suggest that GDF15 contributes to the resistance to 5-FU and OX by promoting cancer stem cell-like properties in CRC. Although GDF15 is classified within the TGF-β superfamily, its signalling activity is not limited to the typical TGF-β/SMAD pathway. Specifically, GDF15 can also activate alternative signalling pathways, including the GFRAL–RET–ERK/AKT, PI3K/AKT, MAPK, and NF-κB pathways [77–80]. These findings indicate that the biological functions of GDF15 extend beyond those of TGF-β itself, suggesting that GDF15 may represent a more selective therapeutic target; however, further investigation is required.

Additionally, high GDF15 expression has been reported as a biomarker associated with metastasis and poor prognosis of CRC [81–83]. We performed immunohistochemical staining of clinical samples from patients with recurrent liver metastases after primary tumour resection, which demonstrated that high GDF15 expression in CRC liver metastases was positively correlated with the rate of second recurrence after resection of recurrent liver metastases and negatively correlated with the time to second recurrence. Furthermore, multivariate analysis identified high GDF15 expression as an independent prognostic factor for second recurrence after hepatic resection. These findings suggest that high GDF15 expression may function as a poor prognostic marker even after recurrence and not only after primary tumour resection, as previously described. In our analysis incorporating the Beppu score, which is a well-established prognostic system for colorectal liver metastasis [27], both the Beppu score and GDF15 expression remained independent predictors of recurrence-free survival. Although patients with high GDF15 expression tended to exhibit elevated Beppu scores, the two variables contributed independent prognostic information, suggesting that GDF15 may reflect tumour biological features that are not fully captured by conventional clinical risk factors. In contrast, we observed no difference in response to chemotherapy between the high- and low-GDF15 groups, likely because we could not reliably evaluate the association between GDF15 expression and response to neoadjuvant chemotherapy in our cohort. This is because only a small proportion of patients received neoadjuvant treatment and there was substantial heterogeneity in chemotherapy regimens and clinical indications, as preoperative chemotherapy is not standardized for resectable colorectal liver metastases [84]. These limitations resulted in markedly variable clinical backgrounds, making it difficult to draw robust conclusions regarding chemotherapy sensitivity in relation to GDF15 expression. Larger and more uniform neoadjuvant cohorts are required to clarify whether GDF15 can serve as a predictive marker of chemotherapy response.

In conclusion, this study provides new insights into the mechanisms underlying drug resistance in CRC cells. Our findings indicate that GDF15 expression contributes to the acquisition of chemoresistance by promoting cancer stemness and is associated with early recurrence and poor clinical outcomes following hepatic resection. Therapeutic interventions targeting GDF15 may provide novel treatment options for patients with CRC with high GDF15 expression.

## Supplementary information

Supplementary Figures and Tables

## Data Availability

The data generated in this study were obtained from the Gene Expression Omnibus (GEO) dataset GSE289766.
