# Supplementary material for: GDF15 promotes 5-Fluorouracil and Oxaliplatin resistance by promoting stem cell-like phenotype in colorectal cancer
Source: Br J Cancer. 2026 May 12;135(4):659–73. doi: 10.1038/s41416-026-03379-0 (PMC13427734; doi:10.1038/s41416-026-03379-0)
Supplement: Supplementary file 1 — Supplementary Figures and Tables [file 41416_2026_3379_MOESM1_ESM.docx]

**Supporting Information:**


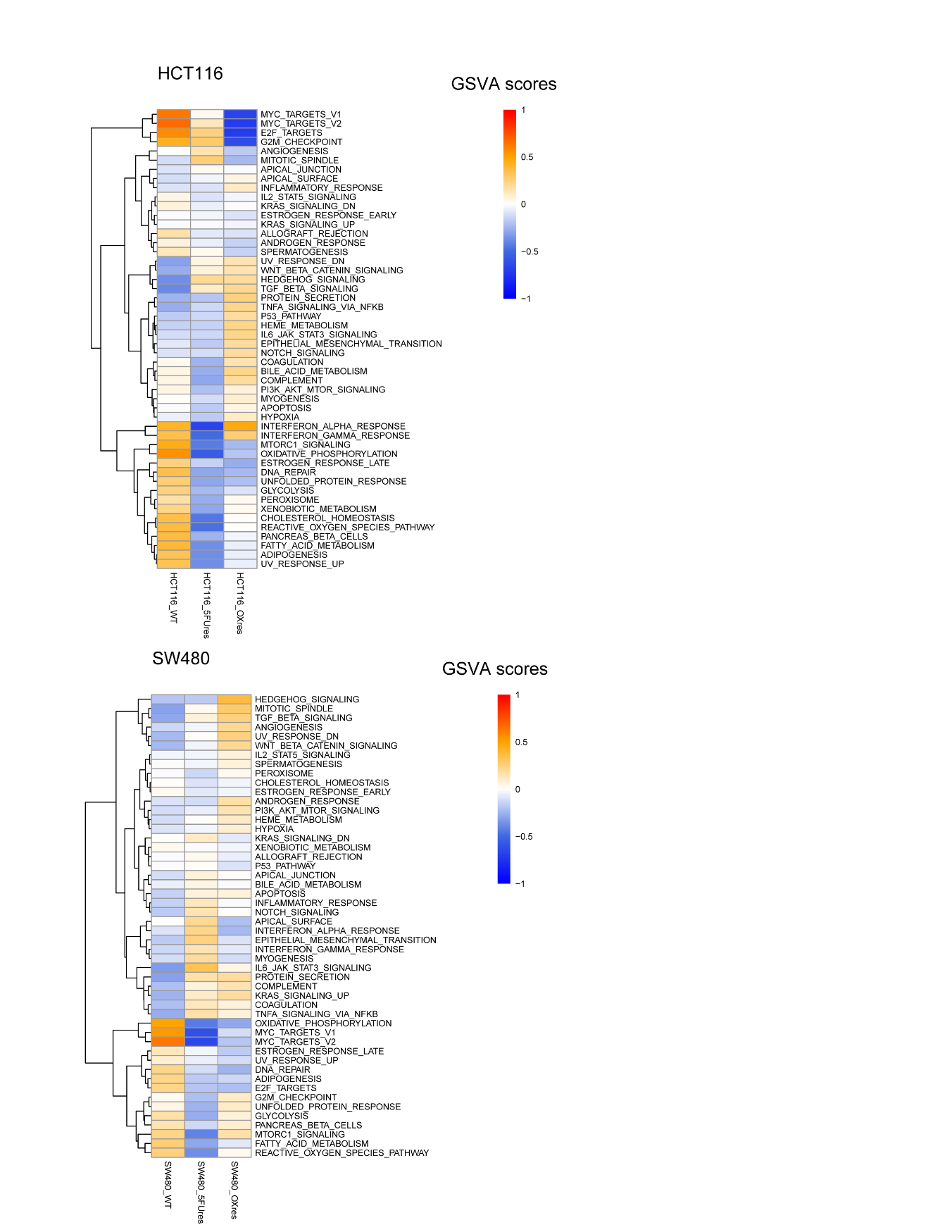


**Supplementary Figure 1. Gene Set Variation Analysis (GSVA) of Hallmark pathway in CRC cells with 5-FU/Oxaliplatin resistant versus control.**

Heatmap of GSVA score in CRC cells with 5-FU/Oxaliplatin resistant versus control.


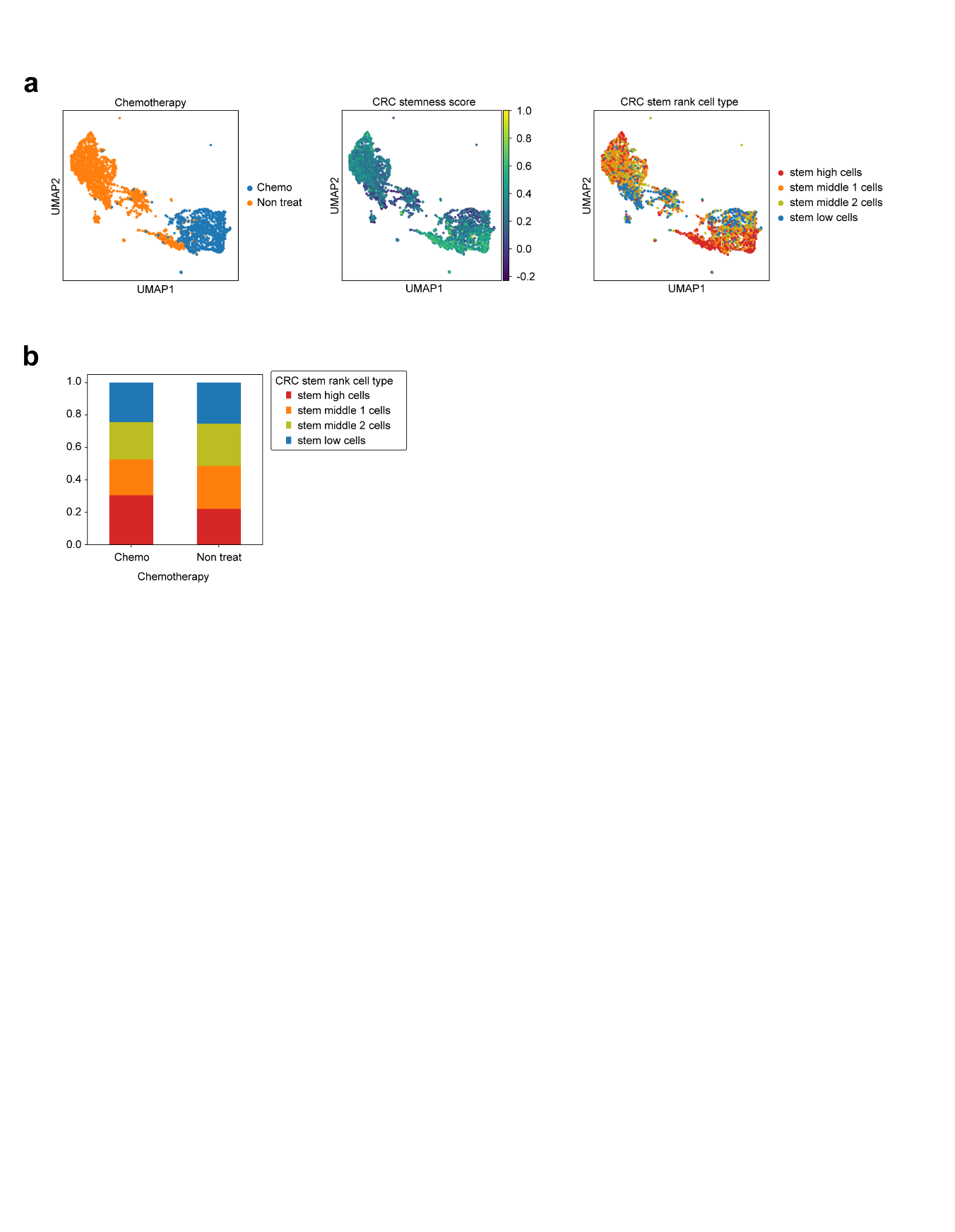
 **Supplementary Figure 2. Analysis of the association between chemotherapy and cancer stemness in single-cell colorectal cancer (CRC) liver metastasis data.**

**a** Uniform Manifold Approximation and Projection (UMAP) of epithelial cells in treatments (left), CRC stemness scores (middle), and CRC stemness rank types (right). **b** Proportion of cells classified based on stemness scores in CRC liver metastatic cells.


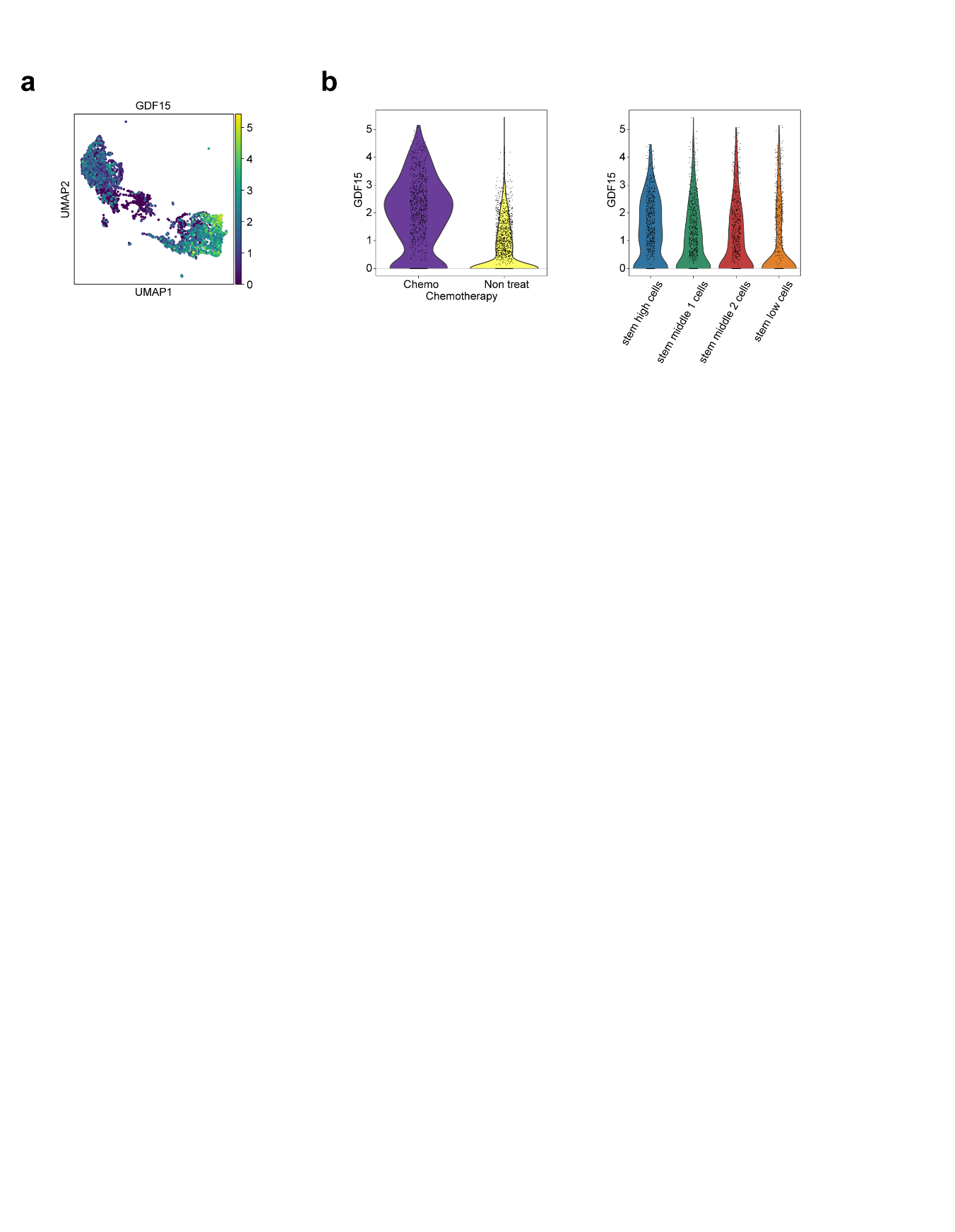
**Supplementary Figure 3. GDF15 expression in single-cell colorectal cancer (CRC) liver metastasis data.**

**a** Uniform Manifold Approximation and Projection (UMAP) of GDF15 expression in epithelial cells. **b** Violin plot of GDF15 expression levels in epithelial cells of treatments (left) and CRC stemness rank types (right).


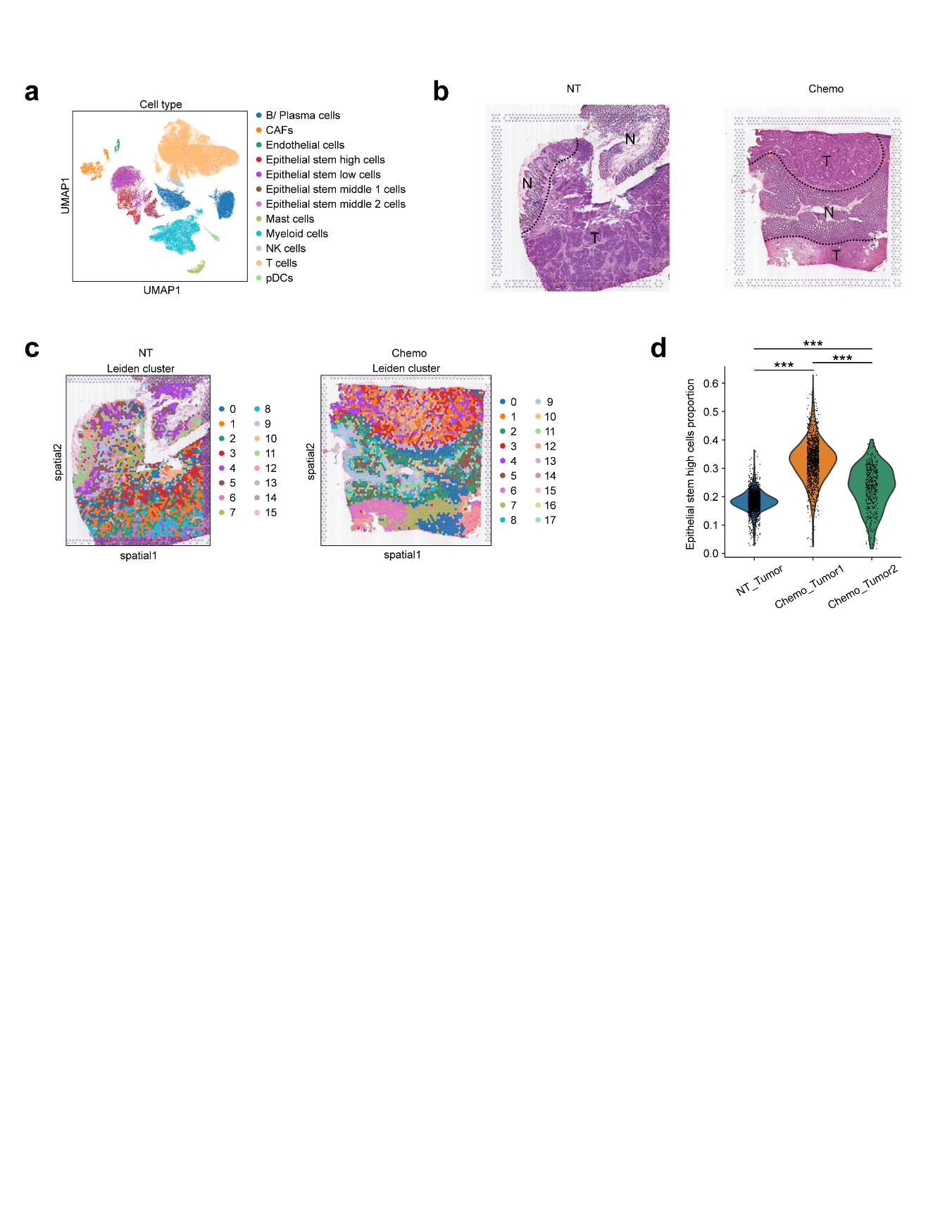


**Supplementary Figure 4. Integrative analysis of spatial transcriptomic and single-cell analysis, including CRC stemness rank types.**

**a** Uniform Manifold Approximation and Projection (UMAP) of all cell types, including CRC stemness rank types. **b** Pathological diagnosis distribution of untreated (NT) and chemotherapy (Chemo) CRC sample slides. **c** Spatial visualization of clusters classified by the Leiden algorithm in the NT and Chemo sample slides. **d** Violin plot representing a high proportion of epithelial stem cells in tumor areas of untreated and chemotherapy-treated CRC samples.


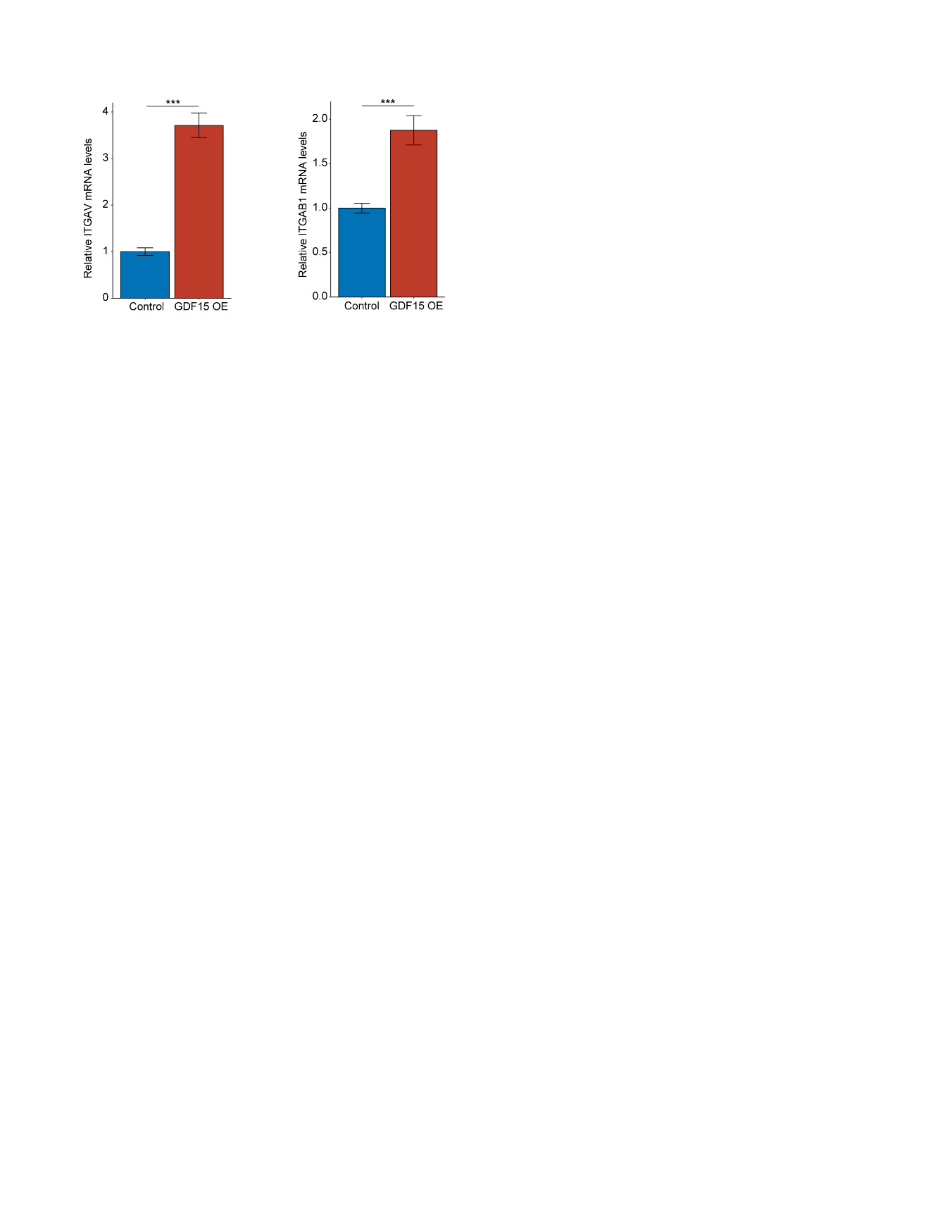
 **Supplementary Figure 5. mRNA expression of cancer stem cell markers using RT-qPCR in GDF15 overexpression colorectal cancer (CRC) cells.**

RT-qPCR analysis of ITGAV and ITGAB1 mRNA expression in HCT116 cells with GDF15 stably overexpression and in control CRC cells. ***P < 0.001; P values were determined using Welch’s t-test.


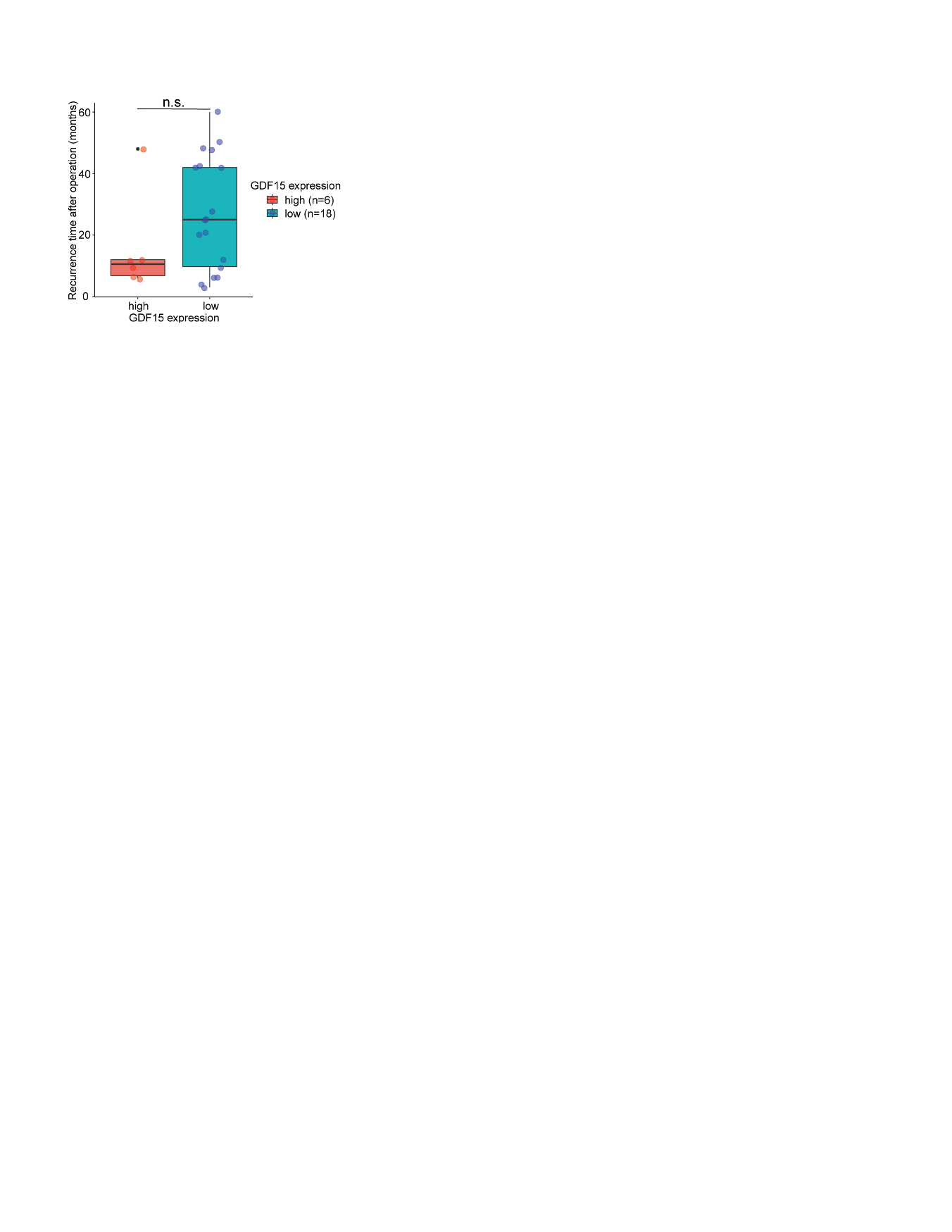


**Supplementary Figure 6. Recurrence time after primary CRC surgery among patients without adjuvant chemotherapy, according to GDF15 expression levels.**

n.s., not significant; *P* values were determined using Welch’s t-test.

**Supplemental Table S1. Primers used for qRT-PCR.**

| Gene | Forward Primer (5'-3')  Reverse Primer (5'-3') |
| --- | --- |
| ACTB | TTAAGGAGAAGCTGTGCTACG  GTTGAAGGTAGTTTCGTGGAT |
| GDF15 | CTCCAGATTCCGAGAGTTGC  AGAGATACGCAGGTGCAGGT |
| KLF4 | CCGCTCCATTACCAAGAGCT  TGGTCAGTTCATCTGAGCGG |
| SOX2 | TACAGCATGTCCTACTCGCAG  GAGGAAGAGGTAACCACAGGG |
| POU5F1 | TTGGGCTAGAGAAGGATGTGGTT  GGAAAAGGGACTGAGTAGAGTGTGG |
| ITGAV | ACCAGGCACTACCGTAAACACA  GGTCCGACCTGGAAAATGCT |
| ITGB1 | ATGTGTCAGACCTGCCTTGG  GCTGGGGTAATTTGTCCCGA |

**Supplementary Table S2. Antibodies used for Simple Western™.**

| Target | Catalog  Number | Host | Supplier |
| --- | --- | --- | --- |
| ACTB | A2006 | Rabbit | Sigma-Aldrich |
| GDF15 | 27455-1-AP | Rabbit | Proteintech |
| KLF4 | 4038 | Rabbit | Cell Signaling  Technology |
| SOX2 | 3579 | Rabbit | Cell Signaling  Technology |
| POU5F1 | 2840 | Rabbit | Cell Signaling  Technology |

**Supplementary Table S3. Correlation between GDF15 expression of liver metastasis tissues and clinicopathological factors among cases who underwent R0 liver resection.**

| Variables | GDF15 high expression (n=25) | GDF15 low expression (n=26) | *P*-value |
| --- | --- | --- | --- |
| Age(≥65y/<65y) | 15/10 | 14/12 | 0.87 |
| Gender (male/female) | 16/9 | 17/9 | 1.00 |
| Adjuvant chemotherapy (+/-) | 19/6 | 8/18 | 0.0031 |
| Size (>2cm/≤2cm) | 15/10 | 18/8 | 0.69 |
| Number (>1/1) | 9/16 | 5/21 | 0.30 |
| Tumor location (rectum/colon) | 6/19 | 9/17 | 0.60 |
| Histological type in liver metastasis  (muc,por,sig /pap,tub) | 3/22 | 0/26 | 0.22 |
| T stage in primary tumor  (is,1,2/3,4) | 5/20 | 7/19 | 0.80 |
| Lymph node metastasis  In primary tumor (+/-) | 20/5 | 6/20 | 0.00015 |
| CEA in liver metastasis resection (>5ng/mL/≤5ng/mL) | 12/13 | 18/8 | 0.21 |
| CA19-9 in liver metastasis resection  (>37U/mL/≤37U/mL) | 14/11 | 10/16 | 0.33 |
| Beppu score  (≥7/<7) | 13/12 | 4/22 | 0.013 |

Pearson's chi-square test; muc, mucinous adenocarcinoma; por, poorly differentiated adenocarcinoma; sig, signet ring carcinoma; pap, papillary adenocarcinoma; tub, tubular adenocarcinoma.

**Supplementary Table S4. Relationship between GDF15 expression in liver metastases and preoperative chemotherapy response.**

|  | Chemotherapy response | | *P*-value |
| --- | --- | --- | --- |
|  | PR/SD (n=20) | PD (n=6) |  |
| GDF15 high expression (Strong, Moderate) | 10 | 3 | 1.00 |
| GDF15 low expression (Weak, Negative) | 10 | 3 |  |

PR, partial response; SD, stable disease; PD, progressive disease.
Chemotherapy response was assessed according to RECIST v1.1 criteria.
